# Cystatin C Trajectories Among Middle-Aged and Older Americans

**DOI:** 10.1093/geroni/igaa057.1661

**Published:** 2020-12-16

**Authors:** Erfei Zhao, Eileen Crimmins, Jennifer Ailshire, Jung Ki Kim, Qiao Wu

**Affiliations:** 1 University of Southern California, Leonard Davis School of Gerontology, Los Angeles, California, United States; 2 University of Southern California, Los Angeles, California, United States; 3 University of Southern California, Los Angeles, United States

## Abstract

Deterioration in kidney functioning is associated with aging and is a major risk factor for mortality and other poor health outcomes. Medicare expenses for poor kidney functioning are about 100 billion dollars every year. High Cystatin-C is an indicator of poor kidney functioning. We do not know if cystatin-C increases gradually as an individual ages. We use the Health and Retirement Study 2006/2008 Biomarker sample with follow-up for 8 years to examine this. Demographic and socioeconomic differences in trajectories of Cystatin-C trajectories were examined for 22,984 participants aged 50 and older. Growth curve models reveal that, although Cystatin-C increases with age (beta=0.025, p<0.001), the annual increase varies by age (60-69 = 0.005, 70-79 = 0.013, 80+ = 0.017, p<0.001), controlling for other socioeconomic variables. Cystatin-C increases faster for males than females. Cystatin-C of non-Hispanic Whites is lower than non-Hispanic Blacks but higher than Hispanics; there is no racial/ethnic difference in change over time. People who spent fewer years in school have higher Cystatin-C, and college graduates have slower growth in Cystatin-C compared to people who did not graduate from high school. These novel findings highlight the disparities in the process of kidney aging among older Americans.

